# Biopsychosocial Impact of Multiple Sclerosis in Omani Patients: A Multicenter Comparative Study

**DOI:** 10.3390/jcm13216315

**Published:** 2024-10-22

**Authors:** Maisaa Al-Dhahri, Mai Helmy, Neeraja Rajeev, Aseel Al Toubi, Hiba Al-Abdali, Abdullah Al-Asmi, Iman Redha Al-Lawati, Issa Al-Adawi, Lakshmanan Jeyaseelan, Samir Al-Adawi

**Affiliations:** 1 Psychiatry Residency Program, Oman Medical Specialty Board, North Azaiba, Muscat 123, Oman; r20122@resident.omsb.org; 2 Department of Psychology, College of Education, Sultan Qaboos University, Al Khoud, Muscat 123, Oman; m.helmy@squ.edu.om; 3 Department of Behavioral Medicine, College of Medicine and Health Sciences, Sultan Qaboos University, Al Khoud, Muscat 123, Oman; neerrajeev@gmail.com (N.R.); a.altoubi@squ.edu.om (A.A.T.); hibaalabdali97@gmail.com (H.A.-A.); 4 Neurology Unit, Department of Medicine, College of Medicine and Health Sciences, Sultan Qaboos University, Muscat 123, Oman; alasm@squ.edu.om; 5 Department of Neurology, Khoula Hospital, Ministry of Health, Minal Al Fahal, Muscat 116, Oman; imanlawati@gmail.com; 6 Department of Sports and Management, Chukyo University, 101 Tokodachi, Kaizu-cho, Toyota 470-0393, Aichi, Japan; j12203m@m.chukyo-u.ac.jp; 7 Basic Science Department, College of Medicine, Mohammed Bin Rashid University of Medicine and Health Sciences, Dubai Healthcare City, Dubai P.O. Box 505055, United Arab Emirates; prof.ljey@gmail.com

**Keywords:** multiple sclerosis, neuropsychological performance, gender differences, quality of life, cognitive functioning

## Abstract

**Background/Objectives**: Multiple sclerosis (MS) is a chronic neurological disorder characterized by various clinical presentations and manifestations that include biopsychosocial impediments. This study has three interrelated goals relevant to biopsychosocial functioning: (i) compare reasoning ability, neuropsychological functioning, affective range, and quality of life (QoL) between people with multiple sclerosis (PwMS) and healthy controls; (ii) explore gender differences in reasoning ability and neuropsychological functioning, affective symptoms, and QoL among PwMS; and (iii) examine the relationship between QoL and cognitive performance in PwMS, focusing on those with inadequate vs. adequate QoL. **Methods**: This multicenter study was carried out among clinically stable PwMS (no relapse in the last two months) at follow-up in two tertiary care units in urban Oman. Healthy controls, matched for age and sex, were also recruited as a comparison group. Data were collected using cognitive batteries sensitive to current reasoning ability and conventional neuropsychological batteries designed to measure verbal learning, visual-spatial ability, and processing speed. The affective range (anxiety and depressive symptoms) and quality of life (QoL) were also evaluated. **Results**: The PwMS group scored lower on current reasoning ability, verbal learning, visual-spatial ability, and processing speed compared to the control group. The incidence of anxiety was higher in the PwMS group, but there were no statistically significant differences in depressive symptoms. No significant differences were found in cognitive variables between the two sexes, except in visual-spatial ability, where women outperformed men. PwMS with low QoL scored lower on attention and concentration indices than those with adequate QoL. According to QoL, no significant differences were observed in reasoning, verbal learning, or visual-spatial ability. **Conclusions**: The present sentinel study suggests that the Omani cohort with MS tends to have lower indices of current reasoning ability, visual and spatial memory, and cognitive speed compared to control subjects. Gender differences are minimal, except for visual-spatial abilities, where women outperform men. Quality of life significantly affects cognitive functioning. In general, the biopsychosocial impediment appears to be significant, indicating the need for comprehensive evaluation and care in the management of MS.

## 1. Introduction

Multiple sclerosis (MS), a chronic autoimmune condition, profoundly impacts both neurological functioning and quality of life, with biopsychosocial factors playing a key role in disease progression and patient well-being. The biopsychosocial model of health recognizes important interactions among biological, psychological, and social factors in disease processes. These interactions include those related to quality of life and adaptive coping [1,2]. Although the biological mechanisms of MS, autoimmune inflammation, demyelination, and axonal injury are well understood, their interaction with psychological and social factors, such as coping mechanisms and quality of life, remains underexplored. In countries in the Arabian Gulf, as well as other Arab populations, MS is a significant health problem, and studies have been carried out to understand its prevalence and risk factors [3,4]. In Saudi Arabia, the prevalence of MS was found to be 40.40 per 100,000 people, and in Dubai, UAE, it was 54.77 per 100,000. Although Oman exhibits a relatively lower prevalence of MS (15 per 100,000) [5] compared to other regional countries (e.g., United Arab Emirates, Kuwait, and Saudi Arabia), Europe and North America, and the worldwide average, understanding the biopsychosocial burdens of PwMS in the country remains crucial given the increasing awareness of cognitive and psychological impairments in global MS populations.

Cognitive or neuropsychological impairment is a common consequence of MS [6]. Cognition involves multiple neural pathways responsible for processing information in the brain. Cognitive impairments can affect multiple domains, including attention, concentration, information processing, executive functioning, visuospatial functions, processing speed, and memory [7]. These cognitive deficits severely limit the daily activities of patients, work performance, and overall autonomy, underscoring the need for a comprehensive cognitive evaluation.

Epidemiological studies estimate that the prevalence of cognitive impairment among PwMS is 40–70% in North America and Europe and 40–60% in Latin America [8,9]. Evaluating cognition in PwMS has become increasingly important, as cognitive functioning directly impacts self-directedness and quality of life. Neuropsychological studies are increasingly used as part of the complete assessment of PwMS. Efforts have been made to establish whether MS is associated with a specific neuropsychological profile [10]. Although an international initiative has made a concerted effort to develop a universally applicable cognitive assessment (Brief International Cognitive Assessment for MS), there are very few studies from the Arab-speaking population [11,12].

Despite the global recognition of cognitive and psychological burdens related to MS, there is a dearth of studies that focus on Arab-speaking populations, particularly those that integrate biological, psychological and social factors. Furthermore, the psychological burden of MS in the context of cognitive functioning has not been adequately explored in this region. Life-limiting diseases such as MS often create a psychosocial burden, including mood disorders, that negatively affects quality of life [13,14,15,16]. The Hospital Anxiety and Depression Scale can be used as a screening tool for mood disorders [17]. Identifying cognitive deficits and psychosocial variables such as mood and quality of life in PwMS has the potential to chart prognostic indicators of MS and lay the foundation for personalized prevention and rehabilitation [18,19,20]. Furthermore, there is also a close relationship between cognition and intellectual capacity [21].

MS has been reported more frequently in women than in men, with a female-to-male ratio of 2.3–3.5:1 [22]. There is a suggestion that this gender disparity in MS has increased in recent times [23]. This has been speculated to be due to ‘nature-nurture’ factors. Alvarez-Sanchez and Dunn have indicated that MS in men tends to have a more significant impairment of cognition and the resulting biopsychosocial impediment compared to women [24]. In support of this view, areas of the brain that critically modulate cognitive functioning are more affected in men than in women [25]. To date, there has been inadequate research that examined gender differences in the currently defined biopsychosocial functioning, such as cognitive, emotional, and quality of life. Understanding these gender differences not only enriches the biopsychosocial framework, but also informs personalized interventions, since male and female patients can experience and respond to disease differently, both cognitively and psychologically.

Although the global prevalence of MS and its associated cognitive and biopsychosocial burdens are well documented, there is a significant gap in research focusing specifically on these aspects in Oman. Oman lacks sufficient studies exploring the integration of biological, psychological, and social factors affecting MS patients, particularly in terms of cognitive and neuropsychological impairments. In Oman, cultural attitudes toward chronic diseases, access to healthcare care, and societal expectations may influence disease experience of the disease and coping strategies. These factors, coupled with variations in healthcare infrastructure, highlight the need for region-specific research that addresses the unique biopsychosocial challenges faced by PwMS in Oman. Therefore, studies are needed to fill this gap by examining the biopsychosocial functioning of Omani PwMS, providing information that could inform customized prevention and rehabilitation efforts in the region. Understanding these dynamics within Oman will help address local healthcare needs and contribute to broader global efforts to manage MS in diverse populations.

To fill the literature gap, this study (i) compares intellectual capacity, neuropsychological functioning, affective range (anxiety and depressive symptoms), and quality of life (QoL) between PwMS and healthy controls; (ii) explores gender differences in current reasoning ability, neuropsychological functioning, and affective functioning among PwMS; and (iii) examines the relationship between quality of life (QoL) and cognitive/neuropsychological performance in PwMS, particularly focusing on those with inadequate vs. adequate QoL.

## 2. Materials and Methods

### 2.1. Study Design and Setting

A multicenter study was conducted among consecutive and clinically stable MS participants at Khoula Hospital and Sultan Qaboos University Hospital from September 2022 to September 2023. These two tertiary care centers have dedicated neurology units that provide specialized care for various neurological conditions, including MS. Oman offers free and universal healthcare to Omanis, and the health system is divided into primary, secondary, and tertiary care. PwMS are referred to two comprehensive tertiary care hospitals [26].

### 2.2. Inclusion Criteria

PwMS who are 18–59 years old with an Expanded Disability Scale (EDSS) score between 0 and 6 at the time of screening were eligible to participate in the study. Clinically stable PwMS were defined as individuals who had not experienced a relapse in the last two months and who were not on corticosteroids during that period.

PwMS were excluded if they had additional neurological or psychiatric disorders, were currently undergoing a relapse, or had used any legal or illegal drugs that could affect cognitive functioning. Further exclusions included PwMS with learning disabilities, poor visual acuity, or those with primary or secondary progressive MS. No exclusions were made based on the duration of MS, but only those with a clear diagnosis of MS such as relapsing-remitting MS, primary progressive MS, secondary progressive MS, and clinically isolated syndrome were included to ensure homogeneity.

The control group consisted of healthy volunteers who matched PwMS in terms of age, sex, and education. Healthy control individuals who did not use psychoactive drugs and had no history of neurological, intellectual or learning disabilities, severe head trauma, or alcohol or drug abuse were enrolled.

### 2.3. Sample Size

Taking into account the expected prevalence of PwMS in Oman (15/100,000), the study included all PwMS who followed up with the Department of Neurology of Sultan Qaboos University and Khoula Hospital, who met the inclusion criteria and consented to participate in the study [5]. Batista et al. have reported that the mean (SD) of general QoL was 58.75 (22.7) units compared to 71.75 (15.11) units in the control group [1]. To estimate this difference with an alpha error at 0.1% level and power at 90% level and expecting a 10% dropout, we needed to study about 100 subjects per group.

### 2.4. Outcome Measures

The outcome measures described in the following include current reasoning ability, neuropsychological assessments, and validated questionnaires to assess psychosocial functioning including variation in the quality of life and mood symptoms/affect. Relevant sociodemographic information and clinical variables were requested directly from the participant or their medical records.

Given the high level of competence required for cognitive and neuropsychological evaluation, research assistants received extensive training from doctorate-level clinical neuropsychologists at Sultan Qaboos University. Six undergraduate psychology students were recruited and underwent rigorous practical training and didactic instruction to administer the outcome measures. The candidates were required to thoroughly familiarize themselves with the practice of the procedure themselves and be evaluated by a senior neuropsychologist to assess their competence. Of the six trainees, only three were considered to have the necessary skills to perform the evaluations for this study.

### 2.5. Current Reasoning Ability

#### Raven Progressive Matrices

Raven’s Colored Progressive Matrices (RPM) is a well-established measure of nonverbal current reasoning ability, intellectual capacity, or intelligence quotient [27]. RPM has 36 items grouped into 3 sets. Each item includes a missing part pattern and several picture inserts. Participants choose the insert that they believe completes the pattern. Raw scores of 36 are converted to percentile scores based on chronological age, as outlined in the RPM manual.

### 2.6. Neuropsychology

#### 2.6.1. Verbal Memory

The California Verbal Learning Test was used to assess short- and long-term verbal memory [28]. The performance of the test has been divided into (i) immediate recall, short-term verbal memory, and (ii) delayed recall, long-term verbal memory. The examiner reads a list of 16 nouns aloud to the participant. Participants were initially asked to recall the words immediately after each presentation of the list. This constitutes a trial that evaluates short-term episodic verbal learning or immediate recall. The second trial, conducted 25 min after the immediate recall test, constitutes a delayed recall and evaluates long-term recall. This test is part of the Brief International Cognitive Assessment for Multiple Sclerosis (BICAMS), a test recommended by an international consortium to develop a specific neuropsychological assessment for PwMS. Alboudi et al. have reported adequate test-retest reliability among Arab-speaking populations in Dubai, UAE [11]. This study used BICAMS to differentiate between PwMS and healthy controls.

#### 2.6.2. Visual-Spatial Ability

The Revised Brief Visuospatial Memory Test was used to assess visual-spatial memory [10]. During the test, participants are presented with a series of geometric figures and are then asked to draw them from memory after being shown to the participant for 10 s [29]. In the literature, participants have responded to verbal responses (i.e., recall/recognition trials) or written responses (i.e., copy trials). This study focused only on written responses. Accuracy is determined by assessing whether the respondent has reproduced the figure and correctly placed it on other figures. This test was also part of BICAMS, and its utility has been reported to be adequate in the Arab-speaking population [11].

#### 2.6.3. Processing Speed

To evaluate the variation in cognitive processing speed, this study used the Symbol Digit Modality Test (SDMT). It requires the participant to match symbols with numbers according to a key, and the score is based on the number of correct matches made within a set time limit. The instructions are to complete the five lines of the task as quickly and accurately as possible in 90 s. The SDMT score is based on the number of correct matches made within the set time limit, with higher scores indicating a faster information processing speed. SDMT has been reported to distinguish between PwMS and healthy controls that matched [11].

### 2.7. Psychosocial Functioning

#### 2.7.1. Affective Range

The affective ranges were evaluated using the Hospital Anxiety and Depression Scale (HADS) by Snaith and Zigmond [30]. HADS is a 14-item checklist of symptoms designed to assess symptoms of anxiety and depression, with 7 items for each. For the present study, the Arabic version of the HADS was used. A cut-off point of 8 is considered to constitute a case of depression or anxiety [31].

#### 2.7.2. Quality of Life

The World Health Organization Quality of Life-Brief Version (WHOQOL-BREF) is a measure of quality of life. The present Arabic version of WHOQOL-BREF has 26 items organized into four subscales: physical health, psychological health, social relationships, and the family environment [32]. The WHOQOL-BREF five-point Likert scale (‘how much’, ‘how completely’, how often, ‘how good’ or ‘how satisfied’). Higher scores denote a better quality of life. For the present purpose and to align with a previous study in Oman, a cutoff point of 60 was used to differentiate those who have ‘adequate’ (>60) or ‘inadequate’ (<60) quality of life [33].

### 2.8. Data Analysis

#### Statistical Methods

Data were analyzed using SPSS 24.0. Continuous outcome variables, that is, intellectual cognitive functioning such as current reasoning ability and short-term verbal memory, etc., were examined for extreme values using the histogram and the Kolmogorov-Smirnov test to test for normality. A chi-square test was performed to compare baseline characteristics such as education, employment, marital status, etc. between MS subjects and the control group. However, the variables of the cognitive functioning outcomes between MS subjects and controls were compared using the appropriate test, such as the paired test or the Wilcoxon signed rank test. The significance level was established at the level of *p* < 0.05.

### 2.9. Ethical Consideration

Participants gave their informed written consent, including their agreement that the accrued data would be anonymized and subsequently published. They were explicitly informed that their participation was voluntary and that they could withdraw at any time with their clinical care remaining unaffected. Ethical approval for the study was obtained from the local Institutional Review Board (IRB) and the Medical Ethics Committee (MREC) of the College of Medicine and Health Sciences, Sultan Qaboos University (Reference No. SQU-EC/590/2021, MREC #2651, approval date: 30 November 2021).

## 3. Results

Table 1 shows the demographic characteristics of PwMS and the control group. In the PwMS group, there were 22 males (21.15%) and 82 females (78.58%). The mean age of PwMS was 36.33 years (SD = 7.99), with a range of 18 to 58 years. Most of the PwMS (74.4%, n = 77) were married, and a significant proportion (46.15%, n = 48) had completed a university or college education, with a bachelor’s degree being the most common level of achievement. Most PwMS (54.81%, n = 57) were employed full-time, and a significant proportion (47.12%, n = 49) worked in white-collar occupations. Most PwMS (84.84%, n = 88) were diagnosed with relapsing-remitting MS, making it the most common subtype of MS.

In the control group, there were 22 men (21.15%) and 82 females (78.58%). The mean age of the control group was 35.61 years (SD = 7.95), with a range of 18 to 58 years. Most of the control group were married (86.54%, n = 90) and a significant proportion had achieved a college or university education (50.96%, n = 53) or a high school diploma (40.38%, n = 42). Approximately 64.42% (n = 67) were employed full-time, with a significant proportion (44.23%, n = 46) working in white-collar occupations.

### 3.1. Aim 1: Compare Reasoning Ability, Neuropsychological Functioning, Affective Range, and Quality of Life Between Patients with Multiple Sclerosis (PwMS) and Controls

As shown in Table 2, 42 PwMS (72.4%) exhibited anxiety symptoms, and 34.8% (n = 35) had depressive symptoms (Hospital Anxiety and Depression Scale). The mean ± SD of the current reasoning ability indices—intellectual quotient (IQ) (Raven Matrices)—that was reported in percentile rank was 29.16 ± 5.18. The mean ± SD for the neuropsychological test was as follows: short-term verbal memory recall (California Verbal Test) = 9.19 ± 3.04; long-term verbal memory recall = 9.86 ± 3.57; visual-spatial ability (Revised Brief Visuospatial Memory Test = 6.52 ± 3.07); and processing speed (Symbol Digit Modality Test) = 29.69 ± 13.37.

In the control group, 27.6% (n = 16) of them had anxiety, and 56.3% (n = 45) had depressive symptoms. The mean ± SD of the current reasoning ability in the percentile scores was 31.56 ± 6.76. The results of the neuropsychological test were the following: short-term verbal memory recall = 11.32 ± 2.88; long-term verbal memory recall = 12.13 ± 2.85; visual-spatial ability = 8.84 ± 2.92) and processing speed = 40.24 ± 13.85).

Table 2 shows the univariate analysis to compare the results of current reasoning ability, neuropsychological batteries, affective functioning, and variation in quality of life between MS patients and the control group. In the PwMS group, the percentile rank was lower in current reasoning ability (t = 8.21, *p* = 0.005). In neuropsychological measures, there was a significant difference in short-term verbal memory recall (t = 26.65, *p* < 0.001), long-term verbal memory recall (t = 25.60, *p* < 0.001), visual-spatial ability: t = 31.17, *p* < 0.001), and processing speed (t = 31.20, *p* < 0.001). For the affective range, the two groups differed significantly in anxiety symptoms (t = 16.16, *p* < 0.0001). There were no statistically significant differences in depression (t = 2.03, *p* = 0.100).

For QoL, the present study used WHOQOL-BREF, which has four subscales: physical health, psychological health, social relationships, and family environment. Only the physical health QoL subscale appeared to differ significantly between PwMS and its counterpart, the control (*p* = 0.005 *).

In summary, in the present comparison between PwMS and the group, there were significant differences in performance across the board except for depressive symptoms, current reasoning ability, three neuropsychological tests (California Verbal Learning Test, Revised Brief Visuospatial Memory Test, and Symbol Digit Modality Test), and anxiety symptoms (subscale of the Hospital Anxiety and Depression Scale). In the QoL indices, only the physical health subscales differed significantly between the two groups.

### 3.2. Aim 2: Analyze Gender Differences in Current Reasoning Abilities, Neuropsychological Test Scores, and Affective Range

Univariate analysis was performed to compare intellectual capacity, cognitive, and affective functioning between PwMS (Table 3). There are no statistically significant differences between men and women in all cognitive variables except visuospatial abilities in which women *outperformed men (Revised Brief Visuospatial Memory Test u = 637.00, p =* 0.034).

### 3.3. Aim 3: Examine the Impact of the Overall Quality of Life (QoL) Score on Current Reasoning Ability and Neuropsychological Batteries

Table 4 shows the univariate analysis of cognitive measures in which PwMS were examined for their QoL status (Inadequate QoL vs. Adequate QoL). PwMS with inadequate QoL showed significantly lower scores on attention and concentration indices than the adequate QoL group (Raven Matrices in percentile rank (T = 294.00, *p* = 0.004)). Furthermore, the difference in processing speed indices (T = 244.00, *p* = 0.016) was also statistically significant. However, statistically significant differences appear between QoL groups in their current reasoning ability, short-term verbal memory (t = 1.27, *p* = 0.205) or long verbal memory (t = 1.55, *p* = 0.124), and visual-spatial ability (t = 1.39, *p* = 0.168).

## 4. Discussion

In Oman, a study by Al-Senani et al. reported a crude prevalence of MS of 15.9 per 100,000 in a facility-based study seeking consultation between 2006 and 2019 [5]. Consequently, Oman constitutes a medium-risk zone for MS. The annual incidence increased from 1.00 to 1.38 cases per 100,000 between 2015 and 2018. The mean age of onset of MS was 27.3 ± 7.7 years and 83% of patients first experienced symptoms between 19 and 40 years. The female-to-male ratio was 2.17:1, and only 9% of the patients had onset of the disease before age 19. To date, this is the first study from an Arab Gulf country to compare neuropsychological and affective functioning between PwMS and a control group to examine whether there are gender differences in neuropsychological performance and to assess how general quality of life affects neuropsychological functioning in PwMS.

During the study period, 104 PwMS were considered to meet the study criteria and recruited along with 104 controls matched for age, sex, and education. Since normative data in the Arab-speaking population for neuropsychological measures have not yet been established, the control group was used as a comparison group, a common practice in neuropsychological research [34]. In the PwMS group, there were 22 men (21.15%) and 82 women (78.58%). The mean age was 36.33 years (SD = 7.99), ranging from 18 to 58 years. Most of the MS patients were married (74.4%, n = 77) and had completed college or university education (46.15%, n = 48), with a bachelor’s degree being the most common. Employment status was largely determined by family income, with 54.81% (n = 57) employed full-time and 47.12% (n = 49) in white-collar occupations. The predominant MS subtype was the relapsing-remitting type (84.84%, n = 88). The preponderance of women and relapsing-remitting subtypes appears to be consistent with previous reports of participants in clinical settings in Oman. These figures echo previous findings of MS patients seeking consultation in the current healthcare setting [3].

### 4.1. Cognitive Performance: Control Versus PwMS

In addition to sociodemographic and clinical risk factors, this study explored whether the cognitive functioning of PwMS differed significantly from that of control participants. Benedict et al. have received the corpus of literature on the prevalence and types of cognitive deficits in people with MS [10]. The prevalence of neuropsychological deficit ranged from 34% to 65% among people with MS, varying by the research setting and the course of the disease. Although only 10% of people tend to have language impediments, semantic memory, and attention span, processing speed deficits appear to be prevalent in 27 to 51% of PwMS, and visual and verbal memory are affected in 54 to 56% and 29–34% of PwMS, respectively. Impairment in executive function indices appears to range from 15 to 28% in PwMS and impairment in visuospatial processing in 22% of PwMS. To date, Paul et al. [12] have conducted a systematic review of cognitive assessment methods for PwMS in the Arab region. The review indicated that cognitive dysfunction is common among PwMS. From the cognitive assessment used, existing data from the Arabic-speaking population appear to suggest that cognitive domains such as memory, attention, and executive function are more likely to be affected. Although data emerging from the Arabic population, as well as from other parts of the world, indicate multi-domain cognitive impairment, there is a significant number of PwMS that exhibit one-domain impairment [35].

The present data showed that PwMS had lower scores on current reasoning ability, verbal learning, memory (both short and long-term), visual-spatial ability, and processing speed than the control group. A point worth pondering is that the indices of current reasoning ability, Raven Matrices, are a test utilized to assess intellectual ability or intellectual quotient (IQ), or specifically, a non-verbal estimate of fluid intelligence. This could ostensibly imply that the current MS cohort is intellectually impaired. It should be noted that none of the participants scored at the impaired level, that is, the percentile ranks below 25. One of the inclusion criteria was adequate intellectual functioning. Therefore, the present data do not show that the MS cohort has an intellectual disability; however, compared to healthy controls, their performance differs significantly. It is also worth noting that Raven Matrices have previously been documented to assess visual-spatial processing and pattern recognition skills, a cognitive domain that has been reported to be commonly affected in people with MS.

### 4.2. Gender and Cognitive Functioning

MS is more prevalent in women, according to a previous study in Oman, and worldwide there is a sex ratio of approximately 2.3 to 3.5:1 [5,36]. The difference between sexes in neuropsychological functioning in PwMS has revealed contradictory results, with data suggesting that women with MS tend to have better cognitive performance compared to men, with less impairment in areas such as memory, attention, and processing speed. Therefore, it appears that women tend to have more ‘brain resilience’ [37]. Many factors could contribute to the difference in brain resilience, including lifestyle, genetic factors, and the health system. Therefore, since most studies come from the ‘global north’, a few from Oman would add a diverse richness to the prevailing debate.

Our results do not show significant gender differences in cognitive variables, except for visual-spatial ability, where women outperformed men. This contradicts established literature, which consistently reports that men tend to outperform women in visuospatial tasks, including critical appraisal such as the meta-analysis reported by Voyer, Voyer and Saint-Aubin [38]. Cognitive impairments may affect men and women differently. To support this, Gavazzi et al. [39] examined sex differences in areas of the brain activated during visuospatial tasks. These authors reported that the activation patterns differ between sexes. Specifically, compared to women, men showed greater activation in the primary visual cortex and the inferior parietal lobe. However, women showed increased activation in the visual cortex compared to men. These findings suggest gender-related variations in brain activation in the processing of visual and spatial items. The second potential reason presented found that sex differences in visual-spatial ability may tend to what is already established in the literature, suggesting that women tend to have a higher cognitive reserve that buffers against some cognitive deficits associated with MS, masking potential differences in areas such as visual-spatial ability [37]. There is some evidence to suggest that hormones such as estrogen have a neuroprotective effect, possibly providing women with some protection against MS-related cognitive decline. This hormonal difference could explain why significant cognitive impairment may not have occurred for women in certain domains.

With increasing recognition that PwMS tends to have various biopsychological impediments, including cognitive impairment, the Brief International Cognitive Assessment for Multiple Sclerosis (BICAMS) has been used to detect specific cognitive impairment of MS [40], which includes various subscales including *the Digit Symbol Modalities Test*, the Brief *Visuospatial Memory Test*, and the *California Verbal Learning Test*, as used in the present study. Additional outcome measures include intellectual ability and mood symptoms, which have been reported to affect cognitive functioning. The present study appears to echo the cognitive profile often reported with BICAMS [40]. Compared to healthy controls, PwMS performed significantly lower on all BICAMS subscales, including verbal learning and recall, visual-spatial ability, and processing speed.

### 4.3. Affective Range

Chronic and debilitating diseases, such as MS, have been well established to be more prone to psychological burden. Psychological effects could arise from several factors, such as the difficulty of living with the disease, the long-term threat of decline and shorter life expectancy, and, in some cases, the stigma associated with the condition [41]. Minden has suggested that, due to the pathological process of demyelination, PwMS are known to exhibit uncontrollable spells of excessive laughter or crying [41]. Various studies have documented in the literature the different types of psychological burden, and it appears that affective ranges, such as symptoms of depression and anxiety, have been examined more frequently [42,43]. Boeschoten et al. have reported a systematic review and meta-analysis on the prevalence of depression and anxiety symptoms in PwMS [14]. These authors identified 58 articles (n = 87,756) on depression and anxiety, and the pooled mean prevalence was 30.5% for depression and 22.1% for anxiety. In Oman, Al-Asmi et al. used the Hospital Anxiety and Depression Scale (HADS) and reported that 35% of the PwMS sample in Oman exhibited anxiety symptoms and 51% depression [31]. However, to date, there are no data on the trajectories of cognitive and neuropsychological symptoms in the expression of anxiety and depressive symptoms. Therefore, cognitive dysfunction is a common occurrence in affective disorders. Cognitive impairment can persist even during periods of symptomatic remission of anxiety or depressive symptoms, and this has been widely documented in various clinical populations [41,44]. However, there is a dearth of studies on the Arab Gulf population. This study examined whether mood disorders influence the neuropsychological profile. Anxiety was found to be higher in PwMS, while depression scores did not show significant differences between the two groups. This appears to echo the literature where anxiety symptoms are common in PwMS. It is not clear whether this endorsement is based on sociocultural teaching. In the Arab Gulf population, distress tends to be expressed through somatic complaints rather than directly reporting emotional or psychological difficulties. This has been hypothesized to be due to cultural patterning, where emotional expression is generally less endorsed. It is also possible that interdependent societies tend to have more social networks due to a tightly knit society, which in turn helps alleviate psychological distress and leads to lower reported levels of depression [45]. However, anxiety symptoms, which often have somatic distress, are more likely to be accepted. Therefore, more research is needed on how MS is experienced in different societies. In relation to this, the study acknowledges the psychological burden associated with MS but does not explore factors such as stigma and social support in detail, which could affect mental health outcomes and their interaction with cognitive performance.

### 4.4. Quality of Life

Several studies have unequivocally suggested that PwMS tends to have a persistent and widespread altered quality of life [46]. Various studies have examined the quality of life among PwMS using various disease-specific scales such as the Multiple Sclerosis International Quality of Life Questionnaire (MusiQoL) and the Multiple Sclerosis Quality of Life Questionnaire (MS-QLQ27) [47]. Although these specific scales have a more heuristic value, to date, most MS disease-specific QoL scales have not been widely validated in the Arab-speaking population. In this sentinel study, the WHOQOL-BREF was used. Although it is not a disease-specific scale for MS, it has been validated in the Arabic-speaking population, and the progenitors of this scale have been specially designed for the cross-cultural population [32,33,48]. There is an indication that quality of life is invariably related to various factors, including age at diagnosis, the presence of physical impairment, mood symptoms, and fatigue [49]. Quality of life is also suggested to be associated with income, education, or general socioeconomic factors. However, PwMS are also affected by neuropsychological deficits and have affected critical areas of the brain involved in cognition [50]. Although QoL itself is strongly dependent on the integrity of cognition, to date there have been a few studies that examined the relationship between neuropsychological functioning and QoL [51]. For brevity, this study has divided QoL into adequate’ (>60) or ‘inadequate’ (>60) QoL as defined by WHOQOL-BREF. The results suggest that PwMS with inadequate QoL is strongly related to current reasoning ability and processing speed compared to those with adequate QoL. No significant differences were observed in the other neuropsychological domains investigated in this study. The present sentinel study would require more robust studies to confirm the present finding. If the present finding could be further scrutiny, it would largely support the view that, in addition to other previously found impediments, PwMS have cognitive impairments that pervade other aspects of biopsychosocial functioning. This echoes John Locke’s view that memory and consciousness are crucial in defining personal identity and self-directedness. Therefore, according to this perspective, intact cognitive functions are essential to maintain one’s sense of identity, a feat that can be challenged by conditions such as MS that impact cognitive abilities.

Despite the limitations mentioned above, the present finding contributes to the existing body of literature on MS by providing region-specific data from Oman, filling a gap in understanding MS in the Arab Gulf population, where research is relatively scarce. First, unlike the global literature, which often shows that men outperform women in visuospatial skills, this study found that Omani women with MS performed better than men in this domain. This contradicts established gender-related cognitive differences in MS and suggests that factors such as increased cognitive reserve in women or neuroprotective effects of hormones (e.g., estrogen) may be relevant in the Omani context. Second, this study confirmed that cognitive impairments, particularly in processing speed, verbal and visual memory, and executive function, are common among PwMS in Oman, reflecting findings from other regions. However, it uniquely shows that these cognitive deficits in the Omani cohort are more likely related to MS pathology than cultural differences, suggesting that cognitive impairment is a global phenomenon in MS patients. Third, this study links cognitive impairments, particularly reasoning ability and processing speed, with poorer quality of life, further strengthening the global understanding that cognitive dysfunction impacts biopsychosocial well-being in PwMS. Fourth, this study highlights a higher prevalence of anxiety but not depression among PwMS in Oman. This contrasts with many global studies that report higher depression rates and underscores the importance of considering sociocultural factors in the expression of psychological distress. The cultural tendency in the Arab Gulf to express emotional distress through somatic complaints may explain this discrepancy, adding a new layer of understanding to how MS affects mental health in different cultural contexts. Overall, this study is the first in the region to compare neuropsychological and affective functioning between PwMS and healthy controls, adding regional data to the global debate on MS-related cognitive impairments.

## 5. Conclusions

This study highlights significant differences in cognitive functioning, neuropsychological performance, and quality of life (QoL) between people with multiple sclerosis (PwMS) and a control group. PwMS demonstrated lower reasoning abilities, poorer performance on neuropsychological tests, and higher levels of anxiety compared to controls. However, there were no significant differences in depressive symptoms. In terms of QoL, the physical health subscale was markedly lower for PwMS. Additionally, gender differences were observed, with women displaying better visual-spatial abilities. The analysis also revealed that PwMS with inadequate QoL scored lower in attention, concentration, and processing speed. In the event of further scrutiny, this sentinel study agrees with other studies in that MS, although marked with variant biological pathological processes, has marked characteristics of biopsychological impediments. Tailored interventions should be considered to support psychosocial challenges, including compromised cognitive functioning and afflictive emotions, in this population.

### Limitations

This study should be considered a sentinel study due to several limitations. The study recruited participants from tertiary centers located in urban areas, which could introduce selection bias. Patients who seek consultation in such facilities may differ from those who do not in ways that could affect the study. To increase statistical stability, the study included various subtypes of MS (relapsing-remitting MS, primary progressive MS, secondary progressive MS, and clinically isolated syndrome). Future studies should include a more homogeneous cohort.

The sample size of the first study, although sufficient for the initial analysis, can limit the generalizability of the findings to all MS patients in Oman or the general population of the Arab world. More studies are needed to confirm these results. Some data, such as quality of life and mood symptoms, were self-reported, which can introduce bias. In addition, such checklists are known to give spurious results compared to gold standard interviews. Participants may underreport or overreport based on personal or social desirability factors. Interestingly, this study revealed a higher incidence of anxiety among PwMS than in the control group. Similarly, performance can differ on the subscale in the indices of psychological, social relationships, and environmental indices in quality of life. Lastly, PwMS are known to use polypharmacy, marked by overt brain atrophy, impaired sleep-wake problems, pathological fatigue, and reduced activity of daily living [52,53]. These, often considered “twin sisters” of MS, tend to affect cognition indirectly. These factors were not explored in this study.

## Figures and Tables

**Table 1:** Characteristics of patients with multiple sclerosis (PwMS) (N = 104) versus control (N = 104).

Characteristics/Demographics		PwMS (n = 104, %)	Control (n = 104, %)	Statistics (*p*-Value)
**Sex**	Male	22 (21.15)	22 (21.15)	1.000 ^a^ (0.567)
	Female	82 (78.85)	82 (78.85)	
**Marital Status**	Single	24 (23.08)	11 (10.58)	7.041 ^a^ (0.071)
	Married	77 (74.04)	90 (86.54)	
	Divorced	2 (1.92)	3 (2.88)	
	Widowed	1 (0.96)	0 (0.0) NA	
**Age**	Mean ± SD	36.33 ± 7.99	35.61 ± 7.95	0.652 ^b^ (0.512)
	Median [Range]	35.50 (18.00–58.00)	35 (18.00–58.00)	
**Age Level**	18:25	7 (6.7)	8 (7.7)	0.852 ^b^ (0.931)
	26:32	24 (23.1)	29 (27.9)	
	33:40	45 (43.3)	40 (38.5)	
	41:48	26 (25.00)	25 (24.00)	
	49:58	2 (1.9)	2 (1.9)	
**Education level**	Below High School	5 (4.81)	8 (7.69)	4.615 ^a^ (0.202)
	High School Diploma	45 (43.27)	42 (40.38)	
	College/University “Bachelor”	48 (46.15)	53 (50.96)	
	Above Bachelor Degree	6 (5.77)	1 (0.96)	2.036 ^a^ (0.565)
**Employment**	Student	4 (3.85)	3 (2.88)	
	Housewife	36 (34.62)	29 (27.88)	
	Unemployed	7 (6.73)	5 (4.81)	
	Employed	57 (54.81)	67 (64.42)	
**Mode of Life/Career**	Professional and Business	16 (15.38)	10 (9.62)	
	Blue Collar	27 (25.96)	35 (33.65)	
	Education and Academia	12 (11.54)	13 (12.50)	
	White collar	49 (47.12)	46 (44.23)	
**Subtypes of PwMS**	Relapsing-Remitting MS	88 (84.62)	NA	
	Primary progressive MS	7 (6.73)	NA	
	Secondary progressive MS	4 (3.85)	NA	
	Clinically isolated syndrome	5 (4.81)	NA	

^a^ Chi-square was used to calculate the significance of differences between samples in categorical variables. ^b^ The Independent Samples *t*-test was used to compare the means of two independent samples. NA, not available.

**Table 2:** Univariate analysis that compares intellectual capacity, neuropsychological functioning, affective range, and quality of life between patients with multiple sclerosis (n = 104) and controls (n = 104).

Intellectual and Cognitive Functioning		MS. Patient (n = 104)	Control (n = 104)	Statistics (*p*-Value)
**Current Reasoning Ability (Raven Matrices in Percentile Scores)**	Mean ± SD	29.16 ± 5.18	31.56 ± 6.76	8.21 ^a^ (0.005 *)
	Median [range]	29.00 (20–45)	29 (19–48)	
**Short-Term Verbal Memory** (**California Verbal Learning Test**)	Mean ± SD	9.19 ± 3.04	11.32 ± 2.88	26.65 ^a^ (<0.0001 **)
	Median [range]	9 (3–16)	12 (4–16)	
**Long-Term Verbal Memory** (**California Verbal Learning Test**)	Mean ± SD	9.86 ± 3.57	12.13 ± 2.85	25.60 ^a^ (<0.0001 **)
	Median [Range]	10 (0–16)	12 (5–16)	
**Visual-Spatial Ability** (**revised Brief Visuospatial Memory Test**)	Mean ± SD	6.52 ± 3.07	8.84 ± 2.92	31.17 ^a^ (<0.0001 **)
	Median [range]	6.25 (0–12)	10 (0–12)	
**Processing Speed** (**Symbol Digit Modality Test**)	Mean ± SD	29.69 ± 13.37	40.24 ± 13.85	31.20 ^a^ (<0.0001 **)
	Median [Range]	30.50 (3–66)	42.00 (6–77)	
**Anxiety** (**Hospital Anxiety and Depression Scale**)	Yes (≥8) No	42 (72.4) 62 (41.3)	16 (27.6) 88 (58.7)	16.16 ^b^ (<0.0001 **)
**Depression** (**Hospital Anxiety and Depression Scale**)	Yes (≥8) No	35 (43.8) 69 (53.9)	45 (56.3) 59 (46.1)	2.03 ^b^ (0.100)
**Quality of Life**	Quality of Life: Physical (low-high)	48 (27.12) 56 (23.43)	69 (37.30) 35 (15.15)	8.61 ^a^ (0.005 *)
	Quality of life: Psychologic (low-high)	46 (25.99) 58 (24.27)	40 (21.62) 64 (27.71)	0.71 ^a^ (0.482)
	Quality of life: Environment (low-high)	38 (21.47) 66 (27.62)	31 (16.76) 73 (31.60)	1.06 ^a^ (0.377)
	Total score of quality of life (low-high)	45 (25.42) 59 (24.69)	45 (24.32) 59 (25.54)	0.00 ^a^ (1.00)

^a^: Z-test; ^b^: *t*-test; *: Sig. at *p* < 0.05; **: Sig. at *p* < 0.001.

**Table 3:** Univariate analysis to compare current reasoning ability, neuropsychological functioning, and affective functioning among people with multiple sclerosis (PwMS) (n = 104).

				Univariate Analysis
Intellectual and Cognitive Functioning		Male PwMS (n = 22)	Female PwMS (n = 82)	Statistics (*p*-Value)
**Current Reasoning Ability (Raven Matrices in Percentile Scores)**	Mean ± SD	28.59 ± 4.97	29.32 ± 5.25	835.50 ^a^ (0.594)
	Median [range]	29.00 (23–45)	29 (20–41)	
**Short-term verbal memory (California Verbal Learning Test)**	Mean ± SD	8.68 ± 2.62	9.33 ± 3.15	801.00 ^a^ (0.419)
	Median [range]	8.50 (4–14)	9 (3–16)	
**Long-term verbal memory (California Verbal Learning Test)**	Mean ± SD	9.00 ± 2.86	10.09 ± 3.72	718.50 ^a^ (0.142)
	Median [range]	10 (3–14)	10 (0–16)	
**Visual-spatial ability (revised Brief Visuospatial Memory Test)**	Mean ± SD	5.39 ± 2.50	6.82 ± 3.15	637.00 ^a^ (0.034 *)
	Median [range]	5 (2–11)	7 (0–12)	
**Processing Speed** (**Symbol Digit Modality Test**)	Mean ± SD	30.77 ± 10.36	29.40 ± 14.11	850.50 ^a^ (0.682)
	Median [range]	30.00 (9–51)	31.00 (3–66)	
**Anxiety** (**Hospital Anxiety and Depression Scale**)	Yes (≥8) No	42 (72.4) 62 (41.3)	16 (27.6) 88 (58.7)	16.16 ^b^ (<0.0001 **)
**Depression** (**Hospital Anxiety and Depression Scale**)	Yes (≥8) No	35 (43.8) 69 (53.9)	45 (56.3) 59 (46.1)	2.03 ^b^ (0.100)

^a^: Mann–Whitney U test; ^b^: statistically significant Mann–Whitney test *, Sig. at *p* < 0.05; **: Sig. at *p* < 0.001.

**Table 4:** Univariate analysis to compare the variation in quality of life (QoL: inadequate vs. inadequate) and current reasoning ability and neuropsychological functioning of MS patients (n = 104).

				Univariate Analysis
Intellectual and Cognitive Functioning		Inadequate QoL (n = 45)	Adequate QoL (n = 59)	Statistics (*p*-Value)
**Current reasoning ability (Raven Matrices percentile scores)**	Mean ± SD	27.58 ± 4.02	30.37 ± 5.65	2.94 ^b^ (0.004 *)
	Median [range]	27.00 (20.00–40.00)	29.00 (23.00–45.00)	
**Short-term verbal memory (California Verbal Learning Test)**	Mean ± SD	8.76 ± 3.09	9.53 ± 2.99	1.27 ^b^ (0.205)
	Median [Range]	9.00 (3.00–16.00)	9.00 (3.00–16.00)	
**Long-term verbal memory (California Verbal Learning Test)**	Mean ± SD	9.22 ± 3.90	10.34 ± 3.25	1.55 ^b^ (0.124)
	Median [range]	10.00 (0.00–16.00)	10 (3.00–16.00)	
**Visual-spatial ability (revised Brief Visuospatial Memory Test)**	Mean ± SD	6.04 ± 2.97	6.88 ± 3.12	1.39 ^b^ (0.168)
	Median [range]	6.00 (0–12)	7.00 (0–12)	
**Processing Speed (Symbol Digit Modality Test)**	Mean ± SD	26.11 ± 12.73	32.42 ± 13.31	2.44 ^b^ (0.016 *)
	Median [range]	24.00 (3–49)	32.00 (3–66)	

QoL (quality of life): adequate QoL (>60) or ‘inadequate’ (<60) QoL as WHOQOL-BREF. ^b^: 2 independent samples—*t*-test; *, Sig. at *p* < 0.05.

## Data Availability

All data generated or analyzed during this study are included in this submission.

## Abbreviations

BICAMS	Brief International Cognitive Assessment for MS
EDSS	Expanded Disability Status Scale
HADS	Hospital Anxiety and Depression Scale
IRB	Institutional Review Board
IQ	Intellectual Quotient
MREC	Medical Research Ethics Committee
MS-QLQ-27	Multiple Sclerosis International Quality of Life Questionnaire
MS	Multiple sclerosis
PwMS	People with Multiple sclerosis
QoL	Quality of life
HADS	Hospital Anxiety and Depression Scale
RPM	Raven’s Progressive Matrices
SQUH	Sultan Qaboos University Hospital
SDMT	Symbol Digit Modality Test
WHOQOL-BREF	World Health Organization Quality of Life—Brief Version
SPSS	Statistical Package for the Social Sciences
